# Association of adiponectin protein and ADIPOQ gene variants with lumbar disc degeneration

**DOI:** 10.3892/etm.2014.1909

**Published:** 2014-08-18

**Authors:** OMAR F. KHABOUR, LAMA ABU-RUMEH, MUHAMMED AL-JARRAH, MOHAMMED JAMOUS, FARAH ALHASHIMI

**Affiliations:** 1 Department of Medical Laboratory Sciences, Faculty of Applied Medical Sciences, Jordan University of Science and Technology, Irbid 22110, Jordan; 2 Department of Rehabilitation Sciences, Faculty of Applied Medical Sciences, Jordan University of Science and Technology, Irbid 22110, Jordan; 3 Department of Neurosurgery, Faculty of Applied Medical Sciences, Jordan University of Science and Technology, Irbid 22110, Jordan

**Keywords:** adiponectin, ADIPOQ, lumbar disc, degeneration, polymorphism

## Abstract

Lumbar disc degeneration (LDD) is a widespread public health problem that may lead to disability and loss of productivity. Adiponectin is an adipokine secreted by adipose tissue and has been shown to be involved in cartilage homeostasis. In the present study, the association between the rs266729 (−11377C/G) and rs2241766 (45T/G) single nucleotide polymorphisms (SNPs) in the adiponectin gene (ADIPOQ) and LDD was investigated. In addition, the correlation between the plasma adiponectin level and LDD was examined. A total of 289 subjects, 168 patients with LDD and 122 healthy individuals, were recruited in the study. All subjects were genotyped for rs266729 and rs2241766 SNPs using polymerase chain reaction-restriction fragment length polymorphism. Circulating levels of adiponectin protein were measured using the ELISA technique. A strong association was found between adiponectin level and LDD (P<0.01), where high levels of adiponectin were found in patients compared with healthy controls. The increase in adiponectin level was not affected by gender. However, no significant differences were found in the genotype distribution or allelic frequency of the two examined polymorphisms between patients with LDD and healthy controls (P>0.05). In conclusion, adiponectin appears to be elevated in patients with LDD. The rs266729 and rs2241766 SNPs in the ADIPOQ gene are not associated with LDD.

## Introduction

Adipose tissue acts as an endocrine organ that secretes several bioactive substances known as adipokines (1). These include leptin, resistin, adiponectin and visfatin that have been shown to play roles in body metabolism, inflammation and susceptibility to diseases (2,3).

Adiponectin is the most abundant adipokine and accounts for ~0.01% of total serum proteins (4). A previous study showed that adiponectin levels are reduced in patients presenting with metabolic syndrome and obesity-related diseases, such as cardiovascular diseases and dyslipidemia (5). However, patients with systemic autoimmune disease exhibit a marked elevation in adiponectin levels (6). At the cartilage level, adiponectin is implicated in the modulation of cartilage destruction in chondrocytes through the adjustment of the activity and levels of inflammatory markers and metalloproteinases (7,8). Therefore, adiponectin is involved in matrix degradation and cartilage homeostasis and may be implicated in diseases that involve cartilage dysfunction. In support of this hypothesis, adiponectin levels are associated with cartilage and bone diseases, including rheumatoid arthritis (RA) (9) and osteoporosis (10–13).

Lumbar disc degeneration (LDD) is a widespread neuromuscular disorder. Recently, several gene variants have been shown to be associated with LDD. For example, genetic variants in collagen types I and II, hypoxia-inducible factor-1α, matrix metalloproteinase 3 and vitamin D receptor genes are associated with LDD (14–19). In the present study, the association between plasma adiponectin levels and LDD was examined. In addition, the association between adiponectin gene (ADIPOQ) variants (rs266729 and rs2241766) and LDD was investigated.

## Materials and methods

### Subjects

A total of 168 patients with LDD were recruited from the King Abdullah University Hospital (Irbid, Jordan) to be involved in the study. In addition, 122 healthy subjects were recruited from the same geographical areas as the patients to serve as the age-matched control group. Patients with RA, systematic lupus erythematosus, osteoporosis and neck pain were excluded from the study. Written informed consent was obtained from each participant according to the requirements of the Jordan University of Science and Technology Institutional Review Board (Irbid, Jordan). Clinical data of the participants were collected from their medical files. Demographic data, including age, gender, body mass index (BMI) and tobacco use, were obtained using a questionnaire.

### Blood collection and handling

Two venous blood samples (3 ml each) in an EDTA tube were obtained from all subjects. One sample was used for DNA extraction and genetic analysis. The other sample was centrifuged at 3,500 × g for 10 min and the plasma was stored at −80°C until use.

### DNA extraction

A DNA isolation kit purchased from Promega Corporation (Madison, WI, USA) was used to extract DNA from all samples according to the standard manufacturer’s instructions. The concentration of the extracted DNA in the samples was determined using a BioRad spectrophotometer (Smart-SpectTM3000; Hemel Hempstead, UK). Samples were stored at −20°C until use.

### Genotyping of ADIPOQ variants

The ADIPOQ gene variants were genotyped using a polymerase chain reaction (PCR)-restriction fragment length polymorphism technique as previously described (20,21). DNA fragments were amplified in PCR tubes containing 2 μM each primer, a ready-to-use master mix (Promega Corporation) and 2 ng DNA. For amplification of the single nucleotide polymorphisms (SNPs), the primer sets were as follows: rs266729, forward (5′-GCT CTG TGT TGG ACT GTG GAG-3′) and reverse (5′-CTG CCA CCC ACT TAG GTG TT-3′); rs2241766, forward (5′-CTG AGA TGG ACG GAG TCC TTT-3′) and reverse (5′-CCA AAT CAC TTC AGG TTG CTT-3′). The PCR amplification conditions comprised denaturation at 94°C for 5 min followed by 35 cycles of 94°C for 60 sec, annealing at 58°C for 35 sec and extension at 72°C for 35 sec, and final extension at 72°C for 7 min. Restriction enzymes used for the genotyping and cleavage conditions were as previously described (20,21). Amplified and digested DNA fragments were visualized using agarose (2%) electrophoresis and ethidium bromide staining.

### Adiponectin serum level

Plasma adiponectin levels were analyzed using an ELISA kit purchased from R&D Systems (DuoSet; Minneapolis, MN, USA). Plasma samples were diluted at 1/800 with the reagent diluent solution supplied by the kit and then 100 μl diluted samples were added into the microplate wells coated with mouse anti-human adiponectin antibody (R&D Systems) (22). The subsequent steps were performed as instructed in the kit. The absorbance of the samples was measured using an ELISA reader (ELx800; BioTek Instruments, Inc., Winooski, VT, USA) at 450 nm and the standard curve was constructed to determine the concentration of samples.

### Measurement of BMI

The body weight and height of each subject were measured in order to determine the BMI, which was calculated using the following formula: BMI = weight (kg)/height (m)^2^.

### Statistical analysis

Data analysis was performed using SPSS version 17.0 (SPSS Inc., Chicago, IL, USA). Genotypes and allele frequencies were evaluated using the χ^2^ test. The Hardy-Weinberg equilibrium of the examined SNPs was also examined using the χ^2^ test. Adiponectin levels are expressed as the mean ± standard error of the mean and were compared between the LDD and control groups using the Student’s t-test. Differences were considered significant at P<0.05.

## Results

### Demographic data

Several demographic parameters were compared between the patient and control groups in the sample (Table I). Baseline data showed no significant differences in age, marital status, physical activity, BMI, family history and smoking habits between the two groups (P>0.05). However, the percentage of females in the patient group (67%) was slightly higher than that in the control group (55%) (P<0.05).

### Association between adiponectin and LDD

**Figure 1:**
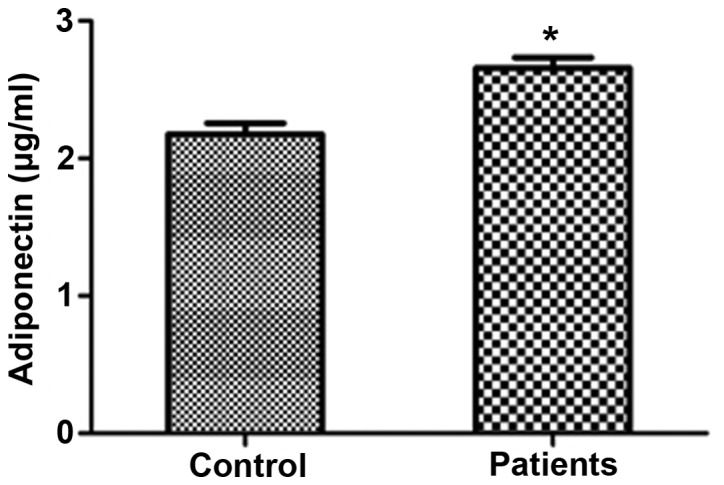
Adiponectin levels in the control and patient groups. Data are presented as the mean ± standard error of the mean. A significant difference in the adiponectin levels was found between the two groups. ^*^Indicates significant difference (P<0.01) from the control group.

**Figure 2:**
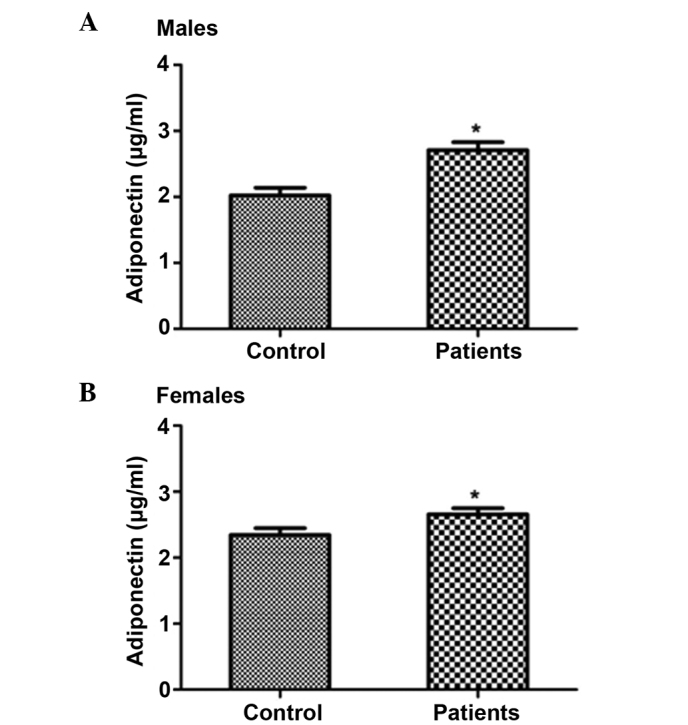
Distribution of adiponectin levels according to gender. Data are presented as the mean ± standard error of the mean. Significant differences in the adiponectin levels were found between the patient and control groups in (A) males and (B) females. ^*^Indicates significant difference (P<0.05) from the control group.

To examine the association between adiponectin and LDD, the plasma adiponectin level was determined using an ELISA technique. The mean plasma adiponectin level in the patient group was 2.659±0.075 μg/ml, while that in the control group was 2.175±0.08 μg/ml (P<0.01). Thus, high adiponectin levels were found to be associated with LDD (Fig. 1). Levels of adiponectin were also analyzed for each gender separately. The association between adiponectin plasma levels and LDD persisted in males (Fig. 2A, P<0.01) and females (Fig. 2B, P<0.05).

### Association between ADIPOQ gene variation and LDD

The association between the variation in the ADIPOQ gene and LDD was also examined. Table II shows the genotypic and allelic distribution of ADIPOQ rs266729 and rs2241766 SNPs in the patient and control groups. The frequency of the T allele of rs2241766 among the control and patients was 73.6 and 79.7%, respectively (P>0.05). With regard to the rs266729 SNP, the frequency of the C allele was 78.6% in the patient group and 82.1% in the control group (P>0.05). Similar to the allele frequencies, the distribution of the different rs266729 and rs2241766 genotypes was similar between the two groups (P>0.05) (Table II). Thus, no significant differences were observed in the genotype and allele frequencies of the examined ADIPOQ gene SNPs between the two groups.

## Discussion

In this study it was shown that adiponectin plasma levels are strongly associated with LDD among adult subjects. The level of plasma adiponectin was higher in patients with LDD than that in the healthy controls.

Adiponectin is a protein specific to adipose tissue that exhibits structural homology to collagens VIII and X and complement factor C1q (23). Adiponectin is rich in the circulation and has been shown to be involved in body metabolism and the immune response (5,6). Recently, adiponectin has been implicated in cartilage and bone diseases (24–26). Several studies have reported elevated adiponectin levels in individuals with RA (9,27–29). The magnitude of the increase in adiponectin levels in RA has been shown to be correlated with disease duration (9). In addition, changes in the baseline adiponectin level have been shown to predict radiographic progression in patients with RA (28). Another bone disease that has been shown to be associated with adiponectin levels is osteoporosis (10–13). A negative association has been reported between adiponectin levels and bone mineral density (13). In animal models, depletion of adiponectin has been shown to protect mice from ovariectomy-induced osteoporosis (30). Finally, a high adiponectin level has been shown to be associated with incident fractures in elderly males (31). In the present study, high plasma adiponectin levels were identified in patients with LDD. Thus, similar to RA, osteoporosis and bone fractures, adiponectin is implicated in the development of LDD. This result adds more evidence to the observed trend of adiponectin elevation in bone and cartilage diseases.

The mechanism by which adiponectin may impact LDD requires investigation. However, a previous study showed that chondrocytes express adiponectin receptors in their membranes (32). In cell cultures, treatment of chondrocytes with adiponectin leads to the activation of the signaling cascade involving adenosine monophosphate-activated protein kinase, c-Jun N-terminal kinase and mitogen-activated protein kinase (MAPK). This leads to activation of the transcription factor nuclear factor-κB and a subsequent elevation in the secretion of matrix metalloproteinases and proinflammatory markers (7,8). Another mechanism by which adiponectin may exert it effects is via the activation of nitric oxide synthase (NOS) inside chondrocytes (8). The activation of NOS increases NO levels inside cells, activates the MAPK signaling pathway and facilitates the release of metalloproteinases from chondrocytes (32), which may lead to cartilage degradation. Thus, changes in adiponectin levels appear to play a role in chondrocyte function and the skeleton, as well as in inflammatory and degenerative cartilage joint diseases.

Previous studies have shown gender differences in the association between adiponectin and certain conditions (20,33–36). For example, elevated adiponectin levels were found to be associated with risk of fracture in older adult males but not in females (33). Adiponectin levels were also shown to be associated with various metabolic risk factors in females but not in males (34). Furthermore, gender differences were found in the association between adiponectin and longevity (20), insulin resistance (35) and BMI (36). In the present study, the observed increase in adiponectin was evident in male and female patients with LDD and did not show any gender differences.

Several studies have indicated that there is an inverse association between adiponectin and obesity (37–39). In the present investigation, no differences were found between patients and healthy subjects in terms of BMI. Thus, the observed higher levels of adiponectin in patients with LDD compared with the control group were not associated with BMI.

In this study, the rs266729 and rs2241766 SNPs in the ADIPOQ gene and their association with LDD were investigated. These SNPs have been shown to affect ADIPOQ gene expression and/or adiponectin activity. In addition, rs266729 and rs2241766 have been shown to be of clinical significance (40–43). The results of the present study showed no association between these SNPs and LDD. Thus, other polymorphisms in the ADIPOQ gene that were not investigated in the present study may be associated with LDD. Since this is the first study to examine the association between ADIPOQ gene SNPs and LDD, to the best of our knowledge, further investigations are required to confirm this result. In conclusion, a strong association was found in the present study between plasma adiponectin level and LDD among patients from Jordan.

## Figures and Tables

**Table I:** Demographic and clinical characteristics of the participants.

Variable	Controls, n (%)	Patients, n (%)	P-value
Age in years^a^	40.46±0.8671	44.14±1.008	0.150
Gender			
Male	56 (45.5)	55 (33.1)	0.032
Female	67 (54.5)	111 (66.9)	
Marital status			
Single	24 (19.5)	26 (15.7)	0.433
Married	99 (80.5)	140 (84.3)	
Physical activity			
Poor	27 (22.0)	32 (19.3)	0.577
Intermediate	17 (13.8)	18 (10.8)	
Good	79 (64.2)	116 (69.9)	
BMI in kg/m^2^			
<18.5	2 (1.6)	1 (0.6)	0.180
18.5–25	38 (30.9)	39 (23.5)	
25–30	44 (35.8)	54 (32.5)	
>30	39 (31.7)	72 (43.4)	
Tobacco use			
Yes	20 (16.3)	19 (11.5)	0.720
No	103 (83.7)	147 (88.5)	

Controls, n=123; patients, n=166.

a Data are presented as the mean ± standard error of the mean.

BMI, body mass index.

**Table II:** Genotype and allele frequencies of rs266729 and rs2241766 in the sample.

Polymorphism	Genotypes and alleles	Controls, n (%)	Patients, n (%)	P-value
rs266729	CC	86 (70.0)	107 (64.4)	0.613
	CG	30 (24.4)	47 (28.3)	
	GG	7 (5.6)	12 (72.3)	
	C allele	202 (82.1)	261 (78.6)	0.483
	G allele	44 (17.9)	71 (21.4)	
rs2241766	TT	111 (66.9)	88 (71.5)	0.441
	TG	37 (22.3)	20 (16.3)	
	GG	18 (10.8)	15 (12.2)	
	T allele	259 (73.6)	196 (79.7)	0.311
	G allele	93 (26.4)	50 (20.3)	

Controls, n=123; patients, n=166.

## References

[1] Fantuzzi G (2005). Adipose tissue, adipokines, and inflammation. J Allergy Clin Immunol.

[2] Conde J, Scotece M, Gómez R (2011). Adipokines: biofactors from white adipose tissue. A complex hub among inflammation, metabolism, and immunity. Biofactors.

[3] Ouchi N, Parker JL, Lugus JJ, Walsh K (2011). Adipokines in inflammation and metabolic disease. Nat Rev Immunol.

[4] Hotta K, Funahashi T, Arita Y (2000). Plasma concentrations of a novel, adipose-specific protein, adiponectin, in type 2 diabetic patients. Arterioscler Thromb Vasc Biol.

[5] Kadowaki T, Yamauchi T, Kubota N (2006). Adiponectin and adiponectin receptors in insulin resistance, diabetes, and the metabolic syndrome. J Clin Invest.

[6] Toussirot E, Gaugler B, Bouhaddi M (2010). Elevated adiponectin serum levels in women with systemic autoimmune diseases. Mediators Inflamm.

[7] Kang EH, Lee YJ, Kim TK (2010). Adiponectin is a potential catabolic mediator in osteoarthritis cartilage. Arthritis Res Ther.

[8] Koskinen A, Juslin S, Nieminen R (2011). Adiponectin associates with markers of cartilage degradation in osteoarthritis and induces production of proinflammatory and catabolic factors through mitogen-activated protein kinase pathways. Arthritis Res Ther.

[9] Rho YH, Solus J, Sokka T (2009). Adipocytokines are associated with radiographic joint damage in rheumatoid arthritis. Arthritis Rheum.

[10] Aǧbaht K, Gürlek A, Karakaya J, Bayraktar M (2009). Circulating adiponectin represents a biomarker of the association between adiposity and bone mineral density. Endocrine.

[11] Doherty AL, Battaglino RA, Donovan J (2014). Adiponectin is a candidate biomarker of lower extremity bone density in men with chronic spinal cord injury. J Bone Miner Res.

[12] Magni P, Dozio E, Galliera E, Ruscica M, Corsi MM (2010). Molecular aspects of adipokine-bone interactions. Curr Mol Med.

[13] Mohiti-Ardekani J, Soleymani-Salehabadi H, Owlia MB, Mohiti A (2014). Relationships between serum adipocyte hormones (adiponectin, leptin, resistin), bone mineral density and bone metabolic markers in osteoporosis patients. J Bone Miner Metab.

[14] Jim JJ, Noponen-Hietala N, Cheung KM (2005). The TRP2 allele of COL9A2 is an age-dependent risk factor for the development and severity of intervertebral disc degeneration. Spine (Phila Pa 1976).

[15] Lin WP, Wang XJ, Wang CR (2013). Polymorphism in the hypoxia-inducible factor 1alpha gene may confer susceptibility to LDD in Chinese cohort. PLoS One.

[16] Näkki A, Videman T, Kujala UM (2011). Candidate gene association study of magnetic resonance imaging-based hip osteoarthritis (OA): evidence for COL9A2 gene as a common predisposing factor for hip OA and lumbar disc degeneration. J Rheumatol.

[17] Seki S, Kawaguchi Y, Mori M (2006). Association study of COL9A2 with lumbar disc disease in the Japanese population. J Hum Genet.

[18] Solovieva S, Lohiniva J, Leino-Arjas P (2006). Intervertebral disc degeneration in relation to the COL9A3 and the IL-1ss gene polymorphisms. Eur Spine J.

[19] Zawilla NH, Darweesh H, Mansour N (2014). Matrix metalloproteinase-3, vitamin D receptor gene polymorphisms, and occupational risk factors in lumbar disc degeneration. J Occup Rehabil.

[20] Khabour OF, Mesmar FS, Alatoum MA, Gharaibeh MY, Alzoubi KH (2010). Associations of polymorphisms in adiponectin and leptin genes with men’s longevity. Aging Male.

[21] Khabour OF, Wehaibi SH, Al-Azzam SI, Alzoubi KH, El-Akawi ZJ (2013). Association of adiponectin with hypertension in type 2 diabetic patients: the gender effect. Clin Exp Hypertens.

[22] Al-Azzam SI, Alkhateeb AM, Alzoubi KH (2013). Atorvastatin treatment modulates the interaction between leptin and adiponectin, and the clinical parameters in patients with type II diabetes. Exp Ther Med.

[23] Nedvidkova J, Smitka K, Kopsky V, Hainer V (2005). Adiponectin, an adipocyte-derived protein. Physiol Res.

[24] Gomez R, Lago F, Gomez-Reino J, Dieguez C, Gualillo O (2009). Adipokines in the skeleton: influence on cartilage function and joint degenerative diseases. J Mol Endocrinol.

[25] Kanazawa I (2012). Adiponectin in metabolic bone disease. Curr Med Chem.

[26] Ruscica M, Steffani L, Magni P (2012). Adiponectin interactions in bone and cartilage biology and disease. Vitam Horm.

[27] Giles JT, van der Heijde DM, Bathon JM (2011). Association of circulating adiponectin levels with progression of radiographic joint destruction in rheumatoid arthritis. Ann Rheum Dis.

[28] Klein-Wieringa IR, van der Linden MP, Knevel R (2011). Baseline serum adipokine levels predict radiographic progression in early rheumatoid arthritis. Arthritis Rheum.

[29] Senolt L, Pavelka K, Housa D, Haluzik M (2006). Increased adiponectin is negatively linked to the local inflammatory process in patients with rheumatoid arthritis. Cytokine.

[30] Wang F, Wang PX, Wu XL (2013). Deficiency of adiponectin protects against ovariectomy-induced osteoporosis in mice. PLoS One.

[31] Johansson H, Oden A, Lerner UH (2012). High serum adiponectin predicts incident fractures in elderly men: Osteoporotic fractures in men (MrOS) Sweden. J Bone Miner Res.

[32] Lago R, Gomez R, Otero M (2008). A new player in cartilage homeostasis: adiponectin induces nitric oxide synthase type II and pro-inflammatory cytokines in chondrocytes. Osteoarthritis Cartilage.

[33] Barbour KE, Zmuda JM, Boudreau R (2011). Adipokines and the risk of fracture in older adults. J Bone Miner Res.

[34] Eglit T, Lember M, Ringmets I, Rajasalu T (2013). Gender differences in serum high-molecular-weight adiponectin levels in metabolic syndrome. Eur J Endocrinol.

[35] Al-Daghri NM, Al-Attas OS, Alokail MS (2011). Gender differences exist in the association of leptin and adiponectin levels with insulin resistance parameters in prepubertal Arab children. J Pediatr Endocrinol Metab.

[36] Tabatabaei-Malazy O, Hasani-Ranjbar S, Amoli MM (2010). Gender-specific differences in the association of adiponectin gene polymorphisms with body mass index. Rev Diabet Stud.

[37] Haluzik M, Parizkova J, Haluzik MM (2004). Adiponectin and its role in the obesity-induced insulin resistance and related complications. Physiol Res.

[38] Hoffstedt J, Arvidsson E, Sjolin E, Wahlen K, Arner P (2004). Adipose tissue adiponectin production and adiponectin serum concentration in human obesity and insulin resistance. J Clin Endocrinol Metab.

[39] Trevaskis JL, Gawronska-Kozak B, Sutton GM (2007). Role of adiponectin and inflammation in insulin resistance of Mc3r and Mc4r knockout mice. Obesity (Silver Spring).

[40] Hashemi M, Hanafi Bojd H, Eskandari Nasab E (2013). Association of adiponectin rs1501299 and rs266729 gene polymorphisms with nonalcoholic fatty liver disease. Hepat Mon.

[41] Hsiao TJ, Wu LS, Huang SY, Lin E (2010). A common variant in the adiponectin gene on weight loss and body composition under sibutramine therapy in obesity. Clin Pharmacol.

[42] Rizk NM, El-Menyar A, Marei I (2013). Association of adiponectin gene polymorphism (+T45G) with acute coronary syndrome and circulating adiponectin levels. Angiology.

[43] Younossi ZM, Jarrar M, Nugent C (2008). A novel diagnostic biomarker panel for obesity-related nonalcoholic steatohepatitis (NASH). Obes Surg.

